# Editorial: The B-Side of B Cells

**DOI:** 10.3389/fimmu.2021.758164

**Published:** 2021-09-03

**Authors:** Alaitz Aranburu, Alessandro Camponeschi, Sven Geissler, Marcella Visentini, M. Manuela Rosado

**Affiliations:** ^1^Department of Rheumatology and Inflammation Research, University of Gothenburg, Gothenburg, Sweden; ^2^Berlin-Brandenburg Center for Regenerative Therapies, Charité-Universitätsmedizin Berlin, Corporate Member of Freie Universität Berlin, Humboldt-Universität zu Berlin, and Berlin Institute of Health, Berlin, Germany; ^3^Department of Translational and Precision Medicine, Sapienza University of Rome, Rome, Italy; ^4^Department of Clinical Internal Sciences, Anesthesiology and Cardiovascular Sciences, Sapienza University of Rome, Rome, Italy

**Keywords:** B cells, IgM natural antibodies, marginal zone (MZ) B cells, IgA, tertiary lymphoid structure, bloodborne infections

B cells play a pivotal role in the humoral immune response by secreting highly diverse but specific antibodies and recently many other B cell functions with important implications on the functional homeostasis of the immune system have been studied.

In this Research Topic we collected new data on the many non-conventional roles of B cells in health and in different disease settings.

Early B cell development and cell lineage commitment occurs in the fetal liver and continue in the bone marrow throughout life after birth. B cells can be subdivided into two main groups (1, 2). B1 cells produce polyreactive “natural” antibodies and are found primarily in the peritoneal and pleural cavities, while B-2 cells produce antigen-induced antibodies in secondary lymphoid organs. Immunoglobulin M (IgM) is the first antibody isotype expressed during development of both cell populations and forms the first humoral B cell receptor (BCR) on the surface of naive B cells. It has previously been assumed that induced IgM antibodies are short-lived and that the corresponding B cells class switch to more effective antibody isotypes. However, recent evidence suggests that IgM antibodies can be produce by long-lived plasma cells and possess memory phenotype with diverse antibody repertoire. How IgM structure and BCR signaling directs B cell development and their responses during infectious and non-communicable diseases is comprehensively reviewed in this issue by Jones et al. Deeper mechanistic insight into fetal B cell development and how it changes in postnatal life is essential for a detailed understanding of the role of B cells during the early onset and progression of disease.

B‐cell acute lymphoblastic leukemia (B‐ALL) is one of the most common cancers in children, with many of the leukemia‐initiating events originating *in utero*. It is likely that the biology of B‐ALL, including leukemia initiation, maintenance and progression depends on the developmental stage and type of B‐lymphoid cell in which it originates. B-ALL is associated with multiple chromosomal translocations that often occur in the fetus and have been differentially associated with poor prognosis and response to treatment. Whether these differences are associated with their emergence in fetal progenitors which express genetic programs that favor self-renewal and proliferation has long been a matter of speculation and driven largely by observations in murine models. The advent of single cell sequencing technology has allowed the transcriptome of hematopoietic cells to be investigated in unprecedented detail. In the mini-review by Jackson et al. the central hypothesis of leukemogenesis is addressed in infant and fetal B cells leukemias which likely result from patterns of genes expressed at the corresponding stages of development. The authors draw their conclusions from recent advances in human fetal lymphoid and B cell progenitors, relevant to understand infant/childhood leukemia, as well as to settle emerging questions in the field (Jackson et al.).

Besides the well-known protective function against invading pathogens, B cells contribute to cellular immunity and regulate or enhance the immune response as antigen presenting cells or effector cells. Many studies have recently attempted to categorize B cells, similar to T cells, into “regulatory” or “effector”, according to their cytokine production profile (3). The homeostasis between regulatory and effector compartments and crosstalk between different cell types are the conditions *sine qua non* for a functional and effective immune response. These multifaceted functions of B cells are currently deeply being investigated in health and diseases that include not only canonical immunological conditions, such as autoimmunity or immunodeficiencies, but also neoplastic, metabolic and neurologic disorders.

In the review by Willsmore et al. the authors discuss the role of B cells in tumor immunity focusing at the melanoma model and how the in deep characterization of B cell subset infiltrating the tumor or generating tertiary lymphoid structures (TLS) is, potentially, a tool to predict not only the response to immune checkpoint inhibitor (CPI) therapy but also tumor progression and metastasization. In fact, whereas infiltrates of regulatory B (Breg) cells may be an indicator of a negative prognosis, intra-tumoral TLS often leads to positive outcome. Generation of anti-tumor specific IgG1 immune responses in TLS potentiates anti-tumor antibody dependent cell-mediated cytotoxic (ADCC) reactions against the tumor by recruiting natural killer cells, cytotoxic T cells, monocytes/macrophages and neutrophils. Although the study of B cell biology in the tumor context is of extreme importance, the key issue that remains is the identification of the factors, present in the tumor microenvironment, that govern B cell fate and thus tumor persistence/survival.

During bacterial invasion the spleen, and particularly marginal zone (MZ) B cells, are key players in controlling blood borne disease by providing the initial round of antibodies. After a bloodborne bacterial infection, neutrophils promptly migrate to the MZ. Lo et al. demonstrated that MZ B cell-deficient mice are more susceptible to systemic *Staphylococcus aureus* infection than wildtype mice. In their article they showed that in the initial phase of infection MZ B cells are able to recruit neutrophils into the marginal zone area in an IL-6/CXCL1/CXCL2 dependent way. The generation of this intimate relation between neutrophils and MZ B cells is crucial for pathogen clearance (Lo et al.).

Interesting to note that same pathogens are able to subvert immune response by means not always clear. As described by Parihar et al., one example is that of *Mycobacterium tuberculosis* bacilli, where formation of tuberculous granulomas are induced, to which B cells actively participate, resulting in persistent infection. The noxious behavior of B cells, in this infection, is partially dependent on IL-4Rα signaling. In fact, mice caring IL-4Rα deficient B cells showed reduced lung pathology to *M. tuberculosis* triggered by the increase, in the lungs, of macrophage pro-inflammatory responses and killing effector functions, and by the local production *M. tuberculosis* specific neutralizing IgA antibodies (Parihar et al.). Translation of this observation into humans will be possible upon the study of B cells isolated from the lungs of infected people.

Hepatitis B virus (HBV) is a non-cytopathic virus, which means that liver damage occurs primarily through the host immune response. Knowledge of the role of B cells has focused primarily on their antibody secretion during the acute phases of the disease. However, recent evidence suggests that they also regulate the immune response beyond antibody secretion (Cai and Yin). The importance of B cells in the immunopathology of a chronic disease progression has long been ignored but has recently gained renewed interest. For example, treatment with the anti-CD20 monoclonal antibody (rituximab) is known to reactivate HBV replication, which can lead to hepatic relapses even in patients with cleared infection (4). In this issue, van Hees et al. investigated the extent to which B-cell activity is related to the different phase of hepatitis B (CHB) infection (5). They profiled the transcriptome of peripheral and intrahepatic B cells between the four clinical phases of CHB infection and healthy controls. Their results showed an important difference between the transcriptomes of intrahepatic and peripheral B cells during HBeAg seroconversion and active regulation of B cell signaling in the liver. The identified unique transcriptome signatures of peripheral and hepatic B cells provide a good resource for studying the microenvironment-dependent effects on B cell immune status.

The transmembrane activator and CAML interactor (TACI) is a receptor encoded by the gene *TNFRSF13B*, and it is crucial for B-cell differentiation and plasma cell survival. Mutations of this gene are often found in common variable immunodeficiency (CVID) and in IgA -deficiency. However, the vast majority of individuals with mutations in *TNFRSF13B* are healthy, with this gene being among the 5% most polymorphic genes in humans. In their review, Cascalho and Platt hypothesize that *TNFRSF13B* diversity might promote innate and adaptive B cell responses.

The role of the immune system in different metabolic disorders such as atherosclerosis and obesity is now emerging as a new field of research with interesting implication in the treatment strategy for these disorders. B cell immunity has been shown to have a particularly important role in atherosclerotic plaque formation and the group of Coleen A. McNamara significantly contributed to the advancement of knowledge in this field (6, 7). In this Research Topic her group shows that chemokine receptor 6 (CCR6) enhances B-1 cell number and IgM secretion in perivascular adipose tissue to provide atheroprotection in mice and demonstrates that in humans, expression of CCR6 on a putative B-1 cell population is significantly reduced in patients with a high degree of coronary artery disease, suggesting that B-1 cell-specific augmentation of CCR6 expression may be a potential therapeutic approach.

The Frasca and Blomberg group analyzes an interesting pro-inflammatory memory B cell subset, called double negative B cells, in patients with obesity and show that these cells are characterized by chronic immune activation, secrete autoantibodies and express the transcription factor T-bet (Frasca et al.). These cells resemble a B cell population found in aged female mice (aged B cells, ABCs) that seem to contribute to a generalized proinflammatory milieu characterizing basal inflammatory states associated with age (8).

Beyond the classical humoral immune responses, or the more familiar A-side, B cells play multiple roles, some of which have been discussed in this Research Topic. We believe that a deeper engagement in the study of the multifaceted functions of B cells as enhancers and regulators of immunity might shed light in the pathogenesis of, not only immune-mediated processes but also metabolic and tumoral diseases, optimizing B cell targeting therapies in different clinical settings.

## Author Contributions

MMR conceptualized the idea of the topic, wrote and revised the manuscript. AA, AC, SG and MV wrote and revised the manuscript. All authors contributed to the article and approved the submitted version.

## Funding

SG was supported by the German Research Foundation (DFG) through funding of the RG2165 (GE2512/2-2) and the CRC1444, the German Federal Ministry of Education and Research (BMBF, 031L0234B) and the European Union’s Horizon 2020 research and innovation programme under grant No 779293 (HIPGEN).

## Conflict of Interest

The authors declare that the research was conducted in the absence of any commercial or financial relationships that could be construed as a potential conflict of interest.

## Publisher’s Note

All claims expressed in this article are solely those of the authors and do not necessarily represent those of their affiliated organizations, or those of the publisher, the editors and the reviewers. Any product that may be evaluated in this article, or claim that may be made by its manufacturer, is not guaranteed or endorsed by the publisher.
